# Relationship Between Severity Classification of Acute Exacerbation of Chronic Obstructive Pulmonary Disease and Clinical Outcomes in Hospitalized Patients

**DOI:** 10.7759/cureus.988

**Published:** 2017-01-22

**Authors:** Pedro J Marcos, Pilar Sanjuán, Arturo Huerta, Irene Nieto-Codesido, Lucía Ferreira-Gonzalez, Oriol Sibila, Marcos I Restrepo

**Affiliations:** 1 Servicio de Neumología. Instituto de Investigación Biomédica de A Coruña (INIBIC), Complejo Hospitalario Universitario de A Coruña (CHUAC), Sergas. Universidade da Coruña (UDC).; 2 Respiratory Medicine, Hospital Lucus Augusti, Lugo; 3 Respiratory Medicine, Hospital Clinic de Barcelona; 4 Respiratory Medicine, CHU A Coruña, Spain; 5 Internal Medicine, Complejo Hospitalario Universitario de Ferrol; 6 Servei de Pneumologia, Hospital Santa Creu i Sant Pau, Barcelona; 7 Department of Medicine, Division of Pulmonary and Critical Care Medicine, South Texas Veterans Health Care System Audie L Murphy VA Hospital and the University of Texas Health Science Center at San Antonio

**Keywords:** copd, exacerbation, hospitalization, guidelines

## Abstract

**Background:**

Limited data are available regarding the impact of the potential validation of the Canadian Thoracic Society (CTS) guidelines recommendations in classifying patients with an acute exacerbation of chronic obstructive pulmonary disease (AECOPD) in simple and complex. The aim of the present study was to assess the CTS recommendations regarding risk stratification on clinical outcomes among patients hospitalized with an AECOPD.

**Methods:**

We developed a retrospective cohort study of patients admitted to one tertiary hospital with a diagnosis of AECOPD. The main clinical outcome was the percentage of treatment failure. Secondary outcomes were 30-day, 90-day, and 1-year readmission and mortality rate, length of stay in hospital, intensive care unit (ICU) admission rate, time to readmission, and time to death. Multivariate analyses were performed using 1-year mortality rate as the dependent measures.

**Results:**

One hundred forty-three patients composed the final study population, most of them (106 [74.1%)] classified as complex acute exacerbation (C-AE) of COPD. C-AE patients had similar rate of treatment failure compared with simple acute exacerbation (S-AE) of COPD (31.1% vs. 27%; p = 0.63). There were no differences regarding the length of stay in hospital, ICU admission rate, and 30-day, 90-day, and 1-year readmission rate. C-AE patients had faster declined measures on time to death (691.6 ± 430 days vs. 998.1 ± 355 days; p = 0.02). In the multivariate analysis, after adjusting for comorbidity, lung function and previous treatment, C-AE patients had a significant higher mortality at one year (Odds Ratio [OR] = 4.9 (Confidence Interval [CI] 95%: 1.16-21); p = 0.031).

**Conclusions:**

In hospitalized patients with an AECOPD, CTS classification, according to the presence of risk factors, was not associated with worse short-term clinical outcomes although it is related with long-term mortality.

## Introduction

Chronic Obstructive Pulmonary Disease (COPD) is a major increasing health problem among people [1]. COPD is currently the fifth most common cause of death in the world, with the World Health Organization (WHO) predicting that it will rank fourth by 2030 [2]. Although there exists certain heterogeneity between patients, the course of COPD is characterized by recurrent acute exacerbations of chronic obstructive pulmonary disease (AECOPD) [3-4]. AECOPD is among the commonest causes of medical admission to hospital and patients with frequent exacerbations have accelerated lung function decline [5], worse quality of life [6-7], and greater mortality [8-9]. Thus, there is a considerable interest in the effectiveness of interventions used both to treat exacerbations and to prevent further events.

Although the infectious agents in COPD exacerbations can be viral or bacterial and the use  of antibiotics in exacerbations still remains controversial [10-12], antibiotics are frequently prescribed in clinical practice. Data from a survey in 360 United States hospitals revealed that 85% of patients admitted for an AECOPD received antibiotics [13]. Similar data have been shown in other countries and with other health services [14]. There is a certain controversy among different clinical practice guidelines related to the administration of antibiotics in AECOPD. Global Initiative for Chronic Obstructive Lung Disease (GOLD) guidelines [15] recommend that antibiotics should be given to patients with three cardinal symptoms (increase in dyspnea, sputum volume, and sputum purulence), with two cardinal symptoms if purulence of sputum is presented, and patients that require mechanical ventilation. Canadian Thoracic Society (CTS) explains in its COPD guidelines [16] that antibiotics are beneficial to treat more severe purulent AECOPD (new increased expectoration of mucopurulent sputum and dyspnea). When choosing antibiotics, CTS guidelines recommend dividing patients in simple or complicated exacerbations, based on the presence of risk factors (RF) that increase the likelihood of treatment failure or are more likely to be associated with virulent or resistant microbial pathogens. However limited data are available regarding the validation of the CTS guidelines recommendations in patients hospitalized with AECOPD. Therefore, we attempt to close these gaps by assessing the CTS recommendations regarding risk stratification on clinical outcomes among patients hospitalized with an AECOPD.

## Materials and methods

### Study population

This is a retrospective cohort study performed in one public hospital in Spain, the Complexo Hospitalario Universitario de A Coruña (CHUAC), a 1382-bed teaching hospital serving a population of 514,466 people. Division permission was obtained in order to collect data regarding impact of the quality improvement initiative at the institution as an exempt study.

Eligible subjects were admitted to the hospital between January 1, 2009 and January 1, 2010 with a hospital discharge diagnosis of AECOPD (International Classification of Diseases 9 [ICD] code 491.20). Subjects were included in the study if they were: (1) >40 years old, (2) former or active smokers (>10 packets per year), and (3) had a prior spirometry with forced expiratory volume at first second/forced vital capacity < 70 (FEV1/FVC < 70). Patients were excluded if they had radiological evidence of pneumonia. If a patient was admitted to the hospital more than once during the study period, only the first hospitalization was recorded and included in the analysis.

### Data abstraction

Chart review data included demographics, comorbid conditions, basal treatment, clinical presentation, laboratory and microbiology data, and treatment during admission. Process measures previously reported, which were associated with higher risk for treatment failure or infection by virulent or resistant microbial pathogens, were recorded. Electronic health records were employed to set the date of readmission and death. Readmission and death were reviewed until 08/01/2013.

### Definitions, AECOPD risk factors, and study groups

Treatment failure was defined as a composite variable of one of these: 30-day readmission, 30-day mortality, the need for intubation, or prolonged length of stay [17]. Prolonged length of hospital stay (LOS) was defined as a length of stay higher than the 75% percentile.

According to CTS COPD guidelines [18], the risk factors associated with a complicated AECOPD are: (1) FEV1 < 50% predicted, (2) ≥4 exacerbations in the last year, (3) ischemic heart disease, (4) use of home oxygen, (5) chronic oral steroid use. If patients had at least one RF at the moment of admission they were considered as a complex acute exacerbation (C-AE), and those without any RF were considered as a simple acute exacerbation (S-AE).

### Clinical outcomes

The main clinical outcome was the percentage of treatment failure. Secondary outcomes were 90-day and 1-year readmission and mortality rate, length of stay in hospital, intensive care unit (ICU) admission rate, time to readmission, and time to death.

### Statistical analysis

Descriptive baseline characteristics were presented as means (± standard deviation [SD]) for continuous variables or n (%) for categorical variables. A two-tailed sample t-test was used to compare the mean length of stay in hospital, time to death, and time to readmission between the study groups. Chi-square test or the Fisher exact test was employed to assess the statistical significance of differences in the rates of participants in the complex and simple acute exacerbation groups who did and did not die, were admitted to the ICU, or were readmitted or died at 30-day, 90-day, or 1-year. We performed a multivariate statistical analysis by logistic regression to assess the independent effect of CTS classification of mortality at one year, with the criteria largest p-value for entering variables (0.05) and smallest p-value for removing variables (0.1). Variables remaining in the multivariate analysis model that showed a p-value of 0.05 were considered significant. Differences among groups in time to death and time to readmission were assessed using the Kaplan-Meier curves and the log-rank test. A two-sided p-value of <0.05 was considered statistically significant in the analysis of proportions. All data were statistically analyzed with SPSS (version 22.0, Chicago, IL, USA) and STATA (version 13 STATA Corporation, College Station, TX, USA).

## Results

**Figure 1:**
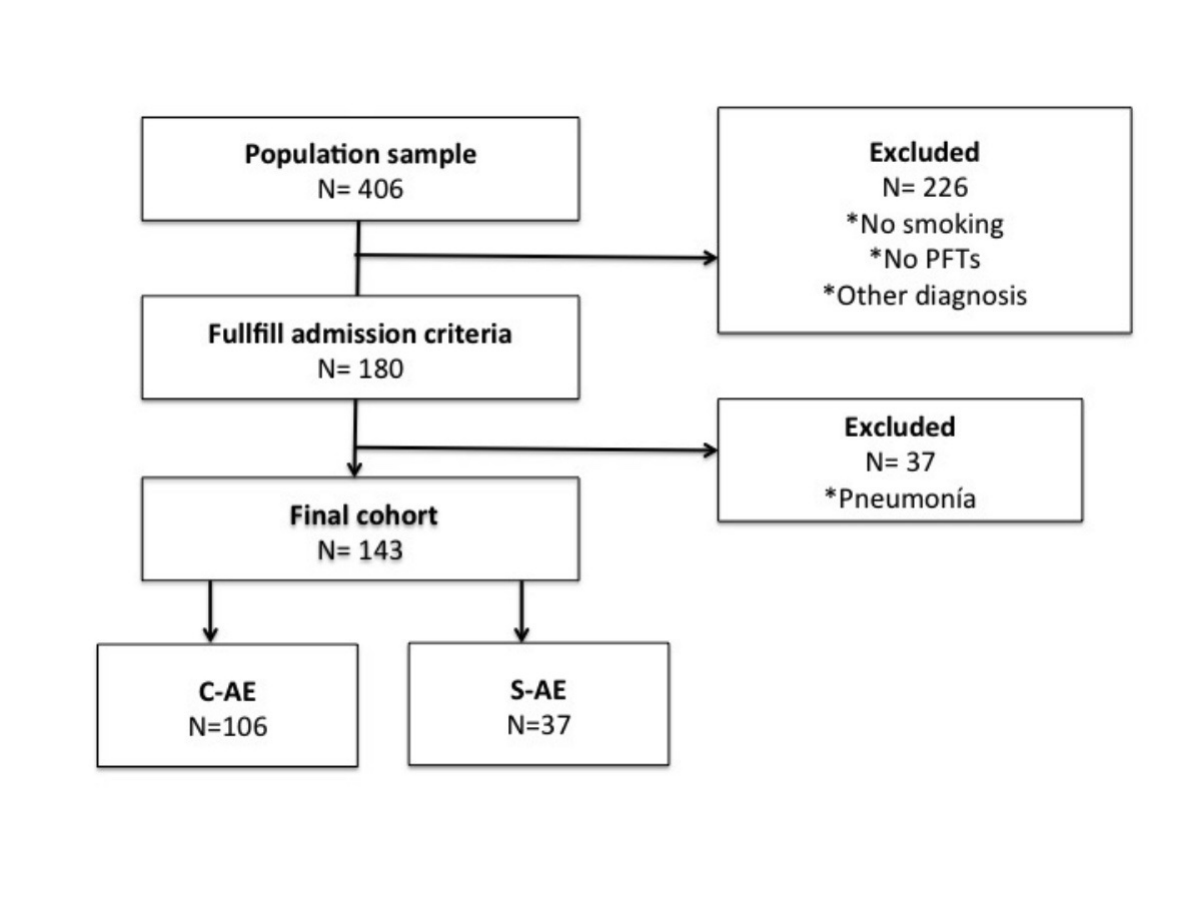
Screening, enrollment, and follow-up of hospitalized patients with acute exacerbation of COPD. C-AE: Complex acute exacerbation; GNB: Gram negative bacilli; S-AE: Simple acute exacerbation.

A total of 406 patients were screened, and among them 226 did not fulfill inclusion criteria (mostly because of the absence of previous lung function test or being nonsmokers) (Figure 1).

A total of 143 patients composed the final study population. Among them 106 patients (74.1%) had at least one risk factor for AECOPD (C-AE group) and 37 patients (25.9%) did not have any risk factor (S-AE group). The proportion of men was significant higher in C-AE group (97.2% vs. 81.1%; p = 0.001).

Demographics, comorbidities, respiratory function, and basal treatment on the admission of the study population are summarized in Table 1.

**Table 1:** Comparison of demographics, comorbidities, pulmonary function, and basal treatment among admitted patients with acute exacerbation of COPD (N = 143) Values are given as n (%), unless otherwise indicated.  ACE: Angiotensin converter enzyme; C-AE: Complex acute exacerbation; FEV1: Forced expiratory volumen at first second; FVC: Forced vital capacity; LABA: Long acting beta agonist; S-AE: Simple acute exacerbation; SD: Standard deviation.

	C-AE (n = 106)	S-AE (n = 37)	p
Demographics			
Men	103 (97.2)	30 (81.1)	0.001
Age	72.17 (SD:9.7)	72.56 (SD:11)	0.849
Current smoker	40 (37.7)	16 (43.24)	0.555
Comorbidities			
Bronchiectasis	16 (15.1)	3 (8.1)	0.216
Previous steroids	10 (9.43)	0 (0)	0.064
Diabetes mellitus	22 (20.7)	2 (5.41)	0.039
Chronic liver disease	10 (9.4)	2 (5.4)	0.73
Hypertension	46 (43.4)	17 (45.9)	0.788
Depression	11 (10.4)	2 (5.4)	0.515
Osteoporosis	5 (4.72)	0 (0)	0.179
Coronary heart disease	19 (17.9)	0 (0)	0.004
Chronic heart failure	23 (21.7)	5 (13.5)	0.28
Peripheral vascular disease	17 (16)	4 (10.8)	0.592
Cerebrovascular disease	9 (8.5)	1 (2.7)	0.454
Dementia	4 (3.8)	1 (2.7)	1
Connective tissue disease	3 (2.8)	0 (0)	0.569
Peptic ulcer	9 (8.5)	4 (10.8)	0.741
Stroke	0 (0)	0 (0)	-
Moderate/severe renal disease	1 (0.9)	0 (0)	1
Tumor	9 (8.5)	3 (8.1)	1
Charlson index	6 (1.7)	5.5 (1.6)	0.0639
Respiratory Function			
FEV/FVC	43.2 (SD:11.7)	54.4 (SD:7.8)	<0.001
FEV1 cc	1122 (SD:395.5)	1691.8 (SD:470.7)	<0.001
FEV1 %	42.5 (SD:15)	67.2 (SD:16.3)	<0.001
FVC cc	2603 (SD:65.9)	2913.5 (SD:765.9)	0.0333
FVC %	75.5 (SD:18.1)	88 (SD:22.4)	0.0032
Basal Treatment			
Tiotropium	80 (75.47)	19 (51.4)	0.006
Ipratropium	12 (11.3)	2 (5.4)	0.297
Salmeterol/Fluticasone	77 (72.6)	12 (32.4)	<0.001
Budesonide/Formoterol	9 (8.5)	3 (8.1)	0.62
LABA monotherapy	1 (0.9)	1 (2.7)	0.45
Inhaled steroids monotherapy	1 (0.9)	2 (5.4)	0.164
Triple therapy	31 (29.2)	2 (62.2)	<0.001
Theophylline	20 (18.9)	0 (0)	0.002
ACE inhibitor	31 (29.2)	6 (16.2)	0.119
Beta blockers	7 (6.6)	4 (10.8)	0.47
Statins	31 (29.2)	9 (24.3)	0.56
Proton pump inhibitors	43 (40.6)	10 (27)	0.142

Excluded by definition that S-AE patients did not have any CTS risk factors, there were no significant differences in the comorbidities between groups, except that C-AE patients had higher frequency of diabetes (20.7% vs. 5.41%; p = 0.039), coronary heart disease (17.9% vs. 0%; p = 0.006)  and a non-significant tendency to have a higher Charlson index (6 ± SD 1.7 vs. 5.5 ± SD 1.6; p = 0.064). As expected by definition, C-AE had worse pulmonary function with a mean FEV1% of 42.5 ± SD 15 vs. 67.2 ± SD 16.3; p > 0.001. Compared with S-AE, C-AE patients were treated before admission more frequently with tiotropium (75.5% vs. 51.5%; p = 0.006), combination of salmeterol/fluticasone (72.6% vs. 32.4%; p < 0.001), triple therapy (tiotropium plus combination long-acting beta agonist/inhaled corticosteroid), and theophylline (18.9% vs. 0%; p = 0.002).

The distribution of CTS risk factors among C-AE is described in Table 2.

**Table 2:** Risk factors associated with a complicated AECOPD according to Canadian Thoracic Society Guidelines Values are given as n (%).

	C-AE (n = 106)
FEV1 < 50%	85 (80.2)
≥4 exacerbations in the last year	11 (10.4)
Ischemic heart disease	19 (17.9)
Use of home oxygen	38 (35.8)
Chronic oral steroid use	10 (9.4)

### Clinical outcomes

C-AE patients had similar rate of treatment failure compared with S-AE (Table 3).

**Figure 2:**
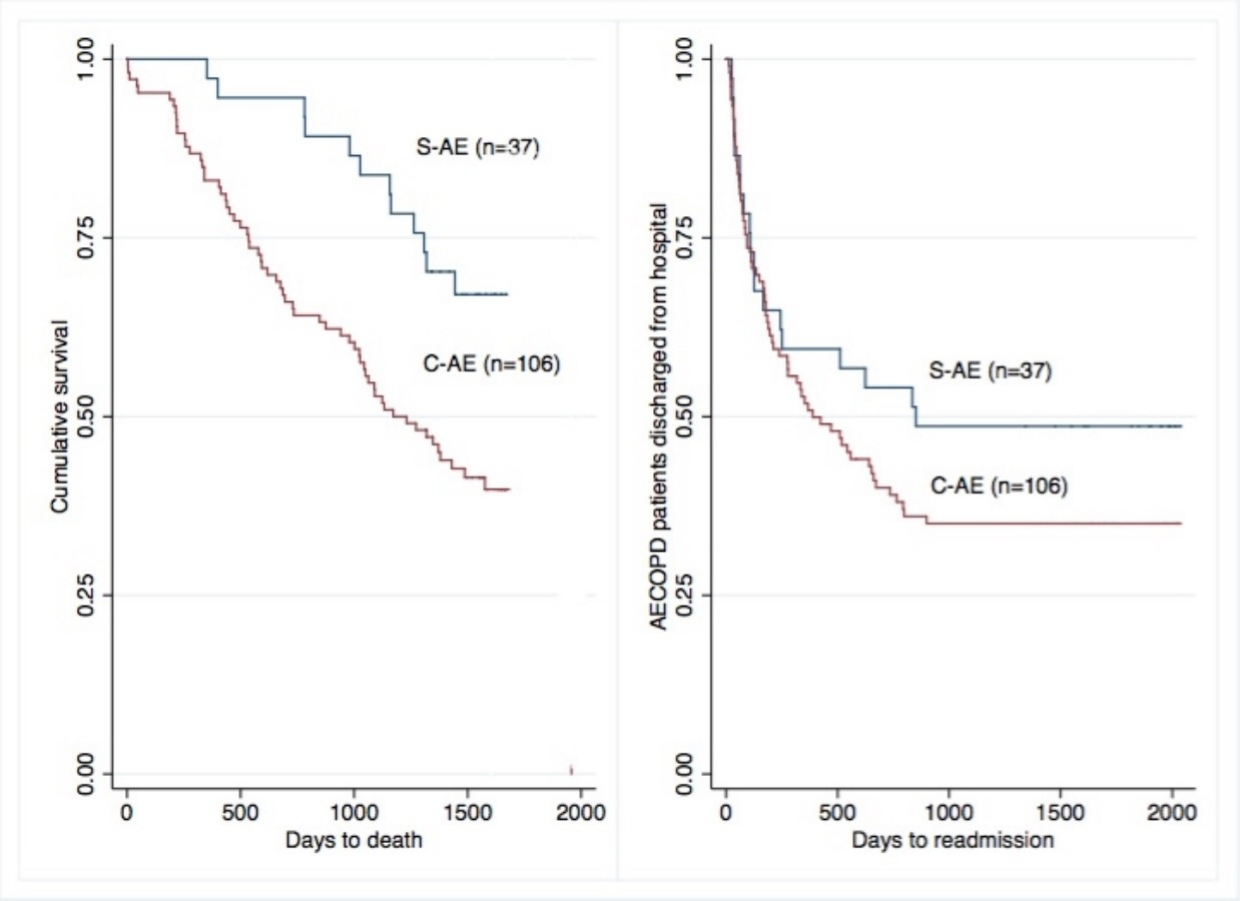
Kaplan–Meier curves of C-AE and S-AE patients. (A) Time to death (p = 0.02). (B) Time to first readmission (p = 0.49). C-AE: Complex acute exacerbation; S-AE: Simple acute exacerbation.

There were no differences between groups regarding length of stay in hospital, ICU admission rate, 30-day, 90-day, and 1-year readmission rate to patients without risk factors. Compared to S-AE, C-AE patients had faster declined measures on time to death (691.6 ± 430 days vs. 998.1 ± 355 days; p = 0.02), without differences in time to first readmission (Table 3) (Figure 2).

**Table 3:** Primary and secondary clinical outcomes for patients in the C-AE and S-AE groups C-AE: Complex acute exacerbation; ICU: Intensive care unit; S-AE: Simple acute exacerbation.

Clinical Outcomes	C-AE (n = 106)	S-AE (n = 37)	p
Treatment failure	33 (31.1)	10 (27)	0.63
30-day readmission	12 (11.3)	5 (13.5)	0.72
90-day readmission	28 (26.4)	8 (21.6)	0.56
1-year readmission	51 (48.1)	15 (40.5)	0.42
30-day mortality	4 (3.77)	0 (0)	0.23
90-day mortality	5 (4.72)	0 (0)	0.18
1-year mortality	18 (17)	1 (2.7)	0.028
Length of stay	9.1 (5.9)	9.4 (4.4)	0.766
ICU admission	4 (3.8)	3 (8.1)	0.375
Time to readmission	240.1 (SD:243.5)	217.4 (SD:272.9)	0.49
Time to death	691.6 (SD:430.4)	998.1 (SD:355.5)	0.02

We found that C-AE patients had a significant higher mortality at one year (Odds Ratio [OR] 4.45, Confidence Interval [CI] 1.16-17]; p = 0.029. In the multivariate analysis and after adjusting for comorbidity, lung function and previous treatment the difference remained significant (OR 4.9 CI 95%: 1.16-21; p = 0.031).

### Complex exacerbation and association with microbial pathogens

Microbiological samples were taken in a similar percentage of patients among each group. Compared with S-AE patients, more sputum cultures were positive among C-AE patients (34.1% vs. 5.9%; p = 0.025). Although virulent or resistant pathogens (Pseudomonas aeruginosa or Stenotrophomonas maltophilia) were more frequently detected in the C-AE patients to detect more, results did not reach statistical significance (Table 4).

**Table 4:** Microbiological diagnosis according to the presence of AECOPD risk factors C-AE: Complex acute exacerbation; S-AE: Simple acute exacerbation. Values are given as No. (%). * : p < 0.05

	C-AE (n = 106)	S-AE (n = 37)
All microbiology collected samples	44 (41.5)	17 (45.9)
Culture positive	15 (34.1)	1 (5.9)*
Streptococcus pneumoniae	1 (6.6)	0 (0)
Haemophilus influenzae	2 (13.3)	0 (0)
Moraxella catarrhalis	2 (13.3)	0 (0)
Pseudomonas aeruginosa	5 (33.3)	1 (100)
Stenotrophomonas maltophilia	5 (33.3)	0 (0)
Escherichia coli	1 (6.6)	0 (0)
MS Staph. Aureus	2 (13.3)	0 (0)
Pseudomonas aeruginosa or Stenotrophomonas maltophilia	9 (60)	1 (100)

## Discussion

Our data showed that CTS COPD acute exacerbation classification in simple and complicated is not a good tool to properly discriminate acute COPD exacerbations in terms of short-term prognosis. However, we detected that simple classification is related with a worse long-term prognosis in terms of mortality.

CTS COPD exacerbation classification [16] was developed to attend the whole COPD population. Since patients with a severe exacerbation who required hospital admission comprised the whole cohort of the study, it is possible that this classification does not work properly in this setting. It is possible that this classification could better work in patients with less severe exacerbations from the outpatient setting. However, there is also a lack of data regarding how this classification impacts in ambulatory COPD exacerbations.

The aim of this classification in simple and complex was to help the clinician when choosing appropriate antibiotics for treating the COPD acute exacerbation. Although antibiotics are recommended in more severe exacerbations associated with purulent sputum, when exacerbation fulfills at least two Anthonisen criteria or when mechanical ventilation is needed [15], real life studies have shown that antibiotics are prescribed in almost all of the patients admitted with a COPD exacerbation [14]. So, tools that could help the clinician to choose when to employ antibiotics and, if possible, which antibiotic to use are really welcome.

We believe that the CTS classification in simple and complex COPD acute exacerbation based only on the detection of at least one risk factor lacks the sensitivity to discriminate severity of COPD exacerbations and to detect worse short-term prognosis, at least in hospitalized patients. Since sicker patients used to have more frequently severe exacerbations requiring hospital admission, it is relatively easy that the patient fulfills at least one of the risk factors which let the patient to be classified as complex (74% in our cohort). So this classification is less useful on the hospital setting leading that almost the exacerbations been classified as complex and therefore to be treated potentially with anti-pseudomonas antibiotics.

On the other hand, we believe that the weight of the variables employed to classify the exacerbations is not the same for each variable. Since low FEV1, to employ oral steroids or having more than ≥4 exacerbations in the last year, have been associated more frequently with Pseudomonas aeruginosa infection [19-20], we do not understand the relation between ischemic heart disease and the complexity of the patients when deciding the use of antibiotics. Maybe this variable could influence a bad outcome related with the severity of the exacerbation but without influence on the microbiologic issue. Another variable is “use of home oxygen.” This variable could represent a more severe disease reflecting the potential risk of an infection with more virulent bacteria; however, we would like to point out that up to that there is a significant percentage of COPD patients where long term oxygen therapy was not correctly prescribed and at the end this variable is reflecting the poor pulmonary function of the patient.

Our study has some limitations that we would like to highlight. First, the retrospective nature of the study: this could lead to an information bias since some confounding factors could be unequally distributed among groups. Second, sputum culture: although it is recommended to all suspected infectious COPD exacerbations, only a low percentage of patients had it; however, this study is reflecting a real life approach when managing hospitalized COPD exacerbations. Third, the population selected is Spanish: we do not know if these CTS recommendations could influence the outcome in the specific Canadian population with a different health system. 

## Conclusions

The CTS classification of AECOPD, according to the presence of risk factors, was not associated with worse short-term clinical outcomes compared to patients without risk factors; however; this classification is a good tool to detect patients with a worse long-term prognosis.
